# The Influence of COVID-19 in Glycemic Control: Predictive Value of Inflammation and Metabolic Parameters

**DOI:** 10.3390/biomedicines12112642

**Published:** 2024-11-19

**Authors:** Minodora Andor, Dana Emilia Man, Daciana Carmen Nistor, Valentina Buda, Simona Dragan

**Affiliations:** 1 Discipline of Medical Semiotics II, Department V—Internal Medicine—1, “Victor Babes” University of Medicine and Pharmacy, 300041 Timisoara, Romania; andor.minodora@umft.ro; 2 Multidisciplinary Heart Research Centre, “Victor Babes” University of Medicine and Pharmacy, 300041 Timisoara, Romania; 3 Department VI—Cardiology, University Clinic of Internal Medicine and Ambulatory Care, Prevention and Cardiovascular Recovery, “Victor Babes” University of Medicine and Pharmacy, 300041 Timisoara, Romania; simona.dragan@umft.ro; 4 Research Centre of Timisoara Institute of Cardiovascular Diseases, “Victor Babes” University of Medicine and Pharmacy, 300041 Timisoara, Romania; 5 Department of Functional Sciences, Physiology, Center of Immuno-Physiology and Biotechnologies (CIFBIOTEH), “Victor Babes” University of Medicine and Pharmacy, 300041 Timisoara, Romania; daciana_nistor@umft.ro; 6 Centre for Gene and Cellular Therapies in Cancer, 300723 Timisoara, Romania; 7 Department I, Faculty of Pharmacy, University Clinic of Clinical Pharmacy, Communication in Pharmacy, Pharmaceutical Care, “Victor Babes” University of Medicine and Pharmacy, 300041 Timisoara, Romania; buda.valentina@umft.ro

**Keywords:** post-COVID-19 diabetes, glycemic outcomes, metabolic factors, HOMA-IR, hs-CRP, TyG index, insulin resistance, predictive analysis, COVID-19 recovery

## Abstract

Background/Objectives: Predicting post-COVID-19 diabetes is crucial for enhancing patient care and public health. This study investigates the role of metabolic factors in predicting the glycemic outcomes in patients recovering from moderate to severe COVID-19. Methods: We conducted a retrospective analysis of 135 patients without pre-existing diabetes, selected from a cohort of 1980 individuals hospitalized between January 2020 and December 2022. Metabolic parameters, including blood glucose, Homeostasis Model Assessment of Insulin Resistance (HOMA-IR), Triglyceride/Glucose (TyG) index, and high-sensitivity C-reactive protein (hs-CRP), were assessed at discharge and followed up after 4 months (T4) and 12 months (T12). Results: Statistical analysis revealed significant correlations of initial glycemia, HOMA-IR, and hs-CRP with the subsequent glycemic levels at T4 and T12. Multiple regression analysis confirmed that initial glycemia, HOMA-IR, and hs-CRP were strong predictors of elevated glycemia, while the TyG index did not show a significant predictive value. Conventional diabetes risk factors, including body mass index (BMI) and lipid profiles, showed low predictive power for post-COVID-19 glycemia. Conclusions: This research highlights the critical role of metabolic and inflammatory pathways in managing glycemic control in COVID-19 patients. Markers like blood glucose, HOMA-IR, and hs-CRP are significant predictors of blood glucose levels, while the TyG index appears less helpful in this context. Early, targeted interventions based on these markers can improve patient outcomes and reduce the risk of post-COVID-19 complications like diabetes.

## 1. Introduction

Predicting the development of diabetes (DM) after COVID-19 is not just valuable but also essential for optimizing patient care, reducing the burden of diabetes-related complications, and improving overall public health outcomes. It allows for proactive management, personalized healthcare, and better resource allocation, ultimately leading to a more effective and efficient healthcare delivery. After the COVID-19 pandemic, numerous studies have demonstrated changes in the metabolic profile, with an increase in the incidence of diabetes. Previous studies indicated that DM is associated with an increased risk of severe COVID-19, acute respiratory distress syndrome (ARDS), and in-hospital mortality [1,2,3]. People with persistent long COVID symptoms can experience pulmonary and extrapulmonary manifestations, including the occurrence of DM. More intriguingly, a recent meta-analysis reported that newly diagnosed DM is frequently seen in patients with COVID-19 [4]. The world has expressed concern about a two-way relationship between these two conditions [5,6]. A follow-up of children with COVID-19 has identified that the incidence of newly diagnosed type 1 DM has increased [7]. Individuals with DM are at greater risk of severe outcomes if they contract COVID-19. This includes increased rates of hospitalization, need for intensive care, and mortality [8].

Additionally, DM can impair the immune response, making it harder for the body to fight off infections, including COVID-19. This can lead to more severe disease progression [9]. Chronic hyperglycemia is associated with increased inflammation, which can exacerbate the inflammatory response seen in severe COVID-19 cases [10]. Some patients who contract COVID-19 have been observed to develop DM even though the classical risk factors for DM are not present. This could be due to the virus’s direct damage to pancreatic beta cells, stress-induced hyperglycemia, or the use of certain medications (e.g., steroids) [11]. Moreover, patients with pre-existing DM may experience severe complications like diabetic ketoacidosis and a hyperosmolar hyperglycemic state when presenting with COVID-19. These conditions often require significantly higher doses of insulin and more intensive medical management [12].

Predicting the development of DM after a COVID-19 infection can be highly useful for several reasons. Identifying individuals at risk of developing post-COVID-19 DM can enable early interventions, such as lifestyle modifications (diet and exercise) and pharmacologic therapy, to prevent or delay the onset of DM. By monitoring individuals identified as at-risk for early signs of hyperglycemia, healthcare professionals can be vigilant and attentive, allowing for timely medical intervention and management. Prediction models can help healthcare providers create personalized treatment plans that address both the acute effects of COVID-19 and the potential for developing DM [13].

Healthcare systems can allocate resources more effectively by identifying patients who may require more intensive follow-up and management. The early detection and management of DM can prevent or reduce the risk of complications such as cardiovascular disease, neuropathy, nephropathy, and retinopathy, which are commonly associated with uncontrolled DM.

Predicting the development of DM can provide valuable data for public health officials to understand the long-term impacts of COVID-19 on population health.

### Purpose of the Study

Experimental observations and data from the literature suggest that blood glucose values, or even the onset of DM, are influenced by both metabolic factors and comorbidities [14,15,16]. In our study, we aimed to investigate these hypotheses by examining the influence of metabolic factors (blood glucose values, HOMA-IR, TyG index) during the acute phase of COVID-19 infection in a group of patients who were subsequently followed up at 4 months and 12 months. Additionally, we considered the comorbidities present before the COVID-19 infection that could contribute to the onset of DM or at least influence elevated glycemic levels.

To make an effective prediction, we used clinical and biological data from a cohort admitted with moderate and severe forms of COVID-19 at the Cardiological Rehabilitation Clinic in Timisoara, Romania.

## 2. Materials and Methods

**Figure 1:**
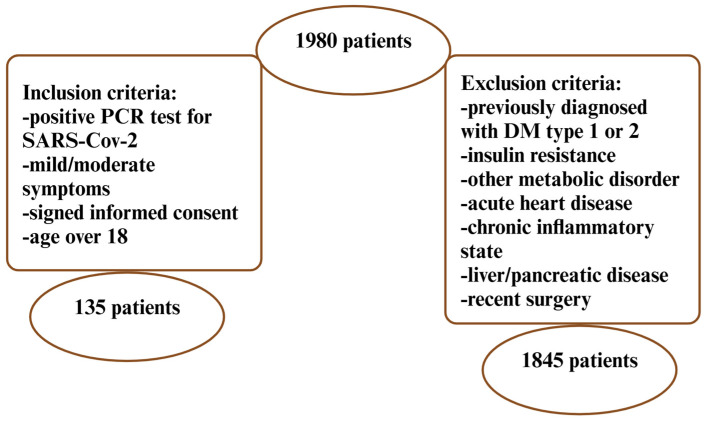
Inclusion/exclusion criteria (created with BioRender.com). Abbreviations: PCR—polymerase chain reaction; SARS-CoV-2—severe acute respiratory syndrome coronavirus 2; DM—diabetes mellitus.

A retrospective study was performed on a cohort of patients hospitalized between January 2020 and December 2022 at the Cardiovascular Rehabilitation Center of the Institute of Cardiovascular Diseases, Timisoara, Romania. The inclusion/exclusion criteria are presented in Figure 1.

### 2.1. Clinical Variables and Laboratory Parameters

Demographic data and epidemiological factors such as gender, age, body mass index, smoking status, hypertension status, or other previous cardiovascular comorbidities, along with clinical and laboratory data, were analyzed. This included an evaluation of cardiovascular risk factors, as well as a review of personal pathological history and comorbidities. Inflammatory marker analysis and the biochemical function test Basic Metabolic Panel (BMP), including the determination of glucose, calcium, electrolytes (Sodium, Potassium, Chloride, Bicarbonate, Blood Urea Nitrogen (BUN) and Creatinine), Estimated Glomerular Filtration Rate (eGFR), Alanine Aminotransferase (ALT) and Aspartate Aminotransferase (AST), Alkaline Phosphatase (ALP), bilirubin, albumin, complete blood count, were carried out. Hemoglobin A1c (HbA1c), insulin levels, HOMA-IR, and TyG index were also analyzed. Fasting serum samples were collected at specific intervals, stored at −80 °C, and tested for proinsulin, HbA1c, and basal blood glucose. Additionally, during the arginine stimulation test and at 4 and 12 months (T0, T4, T12), they were analyzed for insulin, C-peptide, and glucagon. Insulin secretion was measured using an intravenous arginine stimulation test, with blood samples taken at baseline and 2, 5, 10, and 30 min after a 5 g arginine injection. The Acute Insulin Response to Glucose (AIRglucose) was calculated, and beta cell function and insulin resistance were assessed using the insulin-to-proinsulin ratio, HOMA-IR, and TyG index.

Insulin resistance was calculated using the HOMA-IR formula and the Triglyceride/Glucose (TyG) index equation.

HOMA-IR = fasting insulin [(µU/mL) × fasting glucose (mg/dL)]/405(1)

TyG = Ln [TG (mg/dL) × fasting glucose (mg/dL)/2](2)

### 2.2. Statistical Analysis

Our data were stored in an Excel spreadsheet. To assess the influence of metabolic parameters on the prediction of glycemic values post-COVID-19, we performed multiple linear regressions with the dependent variable being glycemia at the 4-month (Glyc-T4) control and at the 12-month (Glyc-T12) control. The significance level for the correlation coefficient in both simple and multiple linear regression was set at *p* = 0.05. The independent variables were the following metabolic parameters determined during hospitalization for an acute episode of COVID-19, just before discharge when the acute episode was considered cured: blood glucose, Triglyceride/Glucose index (TyG), Homeostasis Model Assessment for Insulin Resistance (HOMA-IR), and high-sensitivity C-reactive protein (hs-CRP).

Since comorbidities prior to COVID-19 infection could have an impact on elevated blood glucose values, another multiple linear regression was performed with the same dependent variables (Glyc-T4 and Glyc-T12) but a different set of independent variables: blood glucose at discharge from the acute episode of COVID-19, body mass index (BMI), waist/hip ratio (W/H), and the lipid profile consisting of the values of total cholesterol (TC), triglyceride (TG), LDL-cholesterol (LDLc), and HDL-cholesterol (HDLc).

### 2.3. Ethics Approval and Patient-Informed Consent

Written informed consent was obtained from all patients for routine admission to our tertiary university hospital for future research and study purposes. Before commencing the study, ethics approval was obtained from the Ethics Committee for Scientific Research of the Cardiovascular Institute of Timisoara (ref. nr. 2193/25 March 2024). The study was carried out following the Declaration of Helsinki.

## 3. Results

The data were processed, leading to the following descriptive statistics results. The blood glucose values are shown in Table 1. The average blood glucose values at T4 and T12 increased compared to the initial average value. The application of the paired t-test showed that these increases were statistically significant (Table 1).

As a first analysis, we examined the pairwise correlations between multiple measurements at discharge and glycemia at T4 and T12. We observed that glycemia at T4 and T12 correlated with initial glycemia, HOMA-IR, and hs-CRP, measured at discharge after the acute episode of COVID-19, with correlation coefficients greater than 0.3 (see Table 2).

We used multiple regression analysis to build a better predictive model of long-term glycemia. We performed multiple regressions predicting glycemia at T4 and T12 with the following independent variables: glycemia at T0, TyG, HOMA-IR, hs-CRP, W/H, BMI, TC, TG, LDLc, and HDLc.

The R value for the T4 prediction was 0.933, while for the T12 prediction, it was 0.886. For the T4 prediction, we observed that three coefficients were significantly predictive: glycemia at T0, HOMA-IR, and hs-CRP. For T12, we observed the same three coefficients being significantly predictive: Glyc-T0, HOMA-IR, and hs-CRP (Table 3).

In a follow-up multiple regression analysis, we examined the dependence of blood glucose at T4 on blood glucose at T0, TyG, HOMA-IR, and hs-CRP. Applying multiple linear correlation analysis led to the results presented in Table 4. A strong multiple correlation was observed. We noted that the parameters of blood glucose at T0, HOMA-IR, and hs-CRP provided strongly significant contributions (all *p* < 0.001), while the contribution of TyG appeared to be insignificant (*p* > 0.05). Similarly, we examined the dependence of the T12 glucose values on the same parameters and observed a very strong multiple correlation. Consistent with the findings from the T4 glucose analysis, the contributions of blood glucose at T0, HOMA-IR, and hs-CRP were significant, while the TyG contribution remained insignificant (*p* > 0.05) (Table 4).

Even though TyG has been reported [17,18] as a reliable surrogate marker of insulin resistance and an independent predictor of DM, our results do not provide evidence for its significance in predicting the evolution of glycemia. If TyG is eliminated from the above table, after recalculations, the results are those in Table 5.

**Figure 2:**
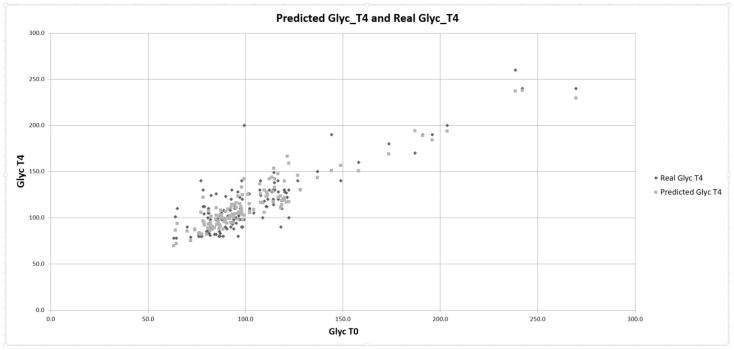
Predicted Glyc-T4 calculated with Equation (3). Glyc-T4—glycemia at the 4-month control; Glyc-T0—initial glycemia.

Hence, we could write the resulting relations for the predicted values pred_Glyc-T4 (Figure 2) and pred_Glyc-T12:

Pred_Glyc-T4 = 17.99 + 0.767 × Glyc-T0 + 1.713 × HOMA + 0.313 × hs-CRP-T0(3)

Pred_Glyc-T12 = 33.56 + 0.618 × Glyc-T0 + 2.082 × HOMA + 0.362 × hs-CRP-T0(4)

A similar plot was used to predict Glyc-T12. A second track mentioned in the study’s hypothesis is the dependence of blood glucose variations on conventional DM risk factors prior to the COVID-19 infection, which could impact changes in blood glucose values. These comorbidities are reflected by W/H, BMI, and lipid profile. Applying multiple linear correlation analyses for T4 blood glucose showed a low correlation coefficient. Similarly, for blood glucose at T12, a low correlation coefficient was found (Table 5). As shown in the tables, none of the coefficients had a statistically significant contribution.

## 4. Discussion

Research shows an increased likelihood of developing DM after COVID-19, especially in the first six months post-infection and in patients with more severe illness [19,20,21,22]. The probability of DM onset appears to increase with the intensity of the infection, with critically ill patients at the highest risk [23,24,25]. According to Xie et al. [21], certain groups, such as elderly individuals, Black patients, and those with conditions like cardiovascular disease, obesity, or hypertension, face a heightened risk of post-COVID-19 diabetes.

Our findings indicated that the blood sugar levels recorded at admission were strong indicators of future glycemic issues, highlighting the need for early blood sugar monitoring in patients hospitalized with COVID-19. Additionally, insulin resistance, measured using HOMA-IR, was a reliable predictor of elevated blood glucose, suggesting that individuals with higher resistance at the onset of illness are more prone to developing hyperglycemia. While the hyperinsulinemic–euglycemic clamp remains the gold standard for evaluating insulin sensitivity, HOMA-IR is widely used for its practicality [26,27,28]. Furthermore, hs-CRP, a marker of inflammation, was identified as a significant predictor of blood sugar imbalances in our study, reflecting the role of inflammation in the metabolic issues seen during COVID-19.

While Geetha et al. [29] have found no correlation between other inflammatory markers like ferritin, lactate dehydrogenase (LDH), and D-dimer with new-onset hyperglycemia, our data point to the importance of inflammation in glycemic control during the infection. Elevated hs-CRP levels indicate that the immune response triggered by the virus can interfere with normal glucose metabolism, worsening the condition in patients with COVID-19. Despite previous studies suggesting that the TyG index helps predict type 2 DM, it did not show predictive value for higher blood sugar in our cohort of COVID-19 patients. Park et al. [30] reported that both TyG and HOMA-IR were linked to an increased risk of DM, with TyG often being more accurate for early detection. However, our study found that markers like HOMA-IR and hs-CRP were more effective in predicting glycemic disturbances in COVID-19. Frailty has been associated with an increased risk of hospitalization in diabetic patients, and the combined effects of the SARS-CoV-2 pandemic and type 2 DM have been shown to contribute significantly to frailty. In this context, a study compared clinical and laboratory indices of frail and non-frail diabetic patients during the COVID-19 pandemic, as defined by the Edmonton Frail Scale scores. The results revealed significant differences between the frail and the non-frail groups in terms of blood urea, serum creatinine, eGFR, plasma albumin, TC, TG, HbA1c, mean platelet volume (MPV), and monocyte/lymphocyte ratio (MLR). These findings suggest that frailty in diabetic patients during COVID-19 can be linked to specific metabolic and inflammatory markers, whose determination is essential for managing both DM and frailty in this population [31].

Our research highlights the critical need for early blood sugar monitoring and using indicators such as HOMA-IR and hs-CRP to assess patients at risk of developing glycemic complications during COVID-19. Although the TyG index is valuable in other contexts, it appears less effective in predicting hyperglycemia during COVID-19 than markers like HOMA-IR and inflammation levels. This approach can help healthcare providers better identify at-risk patients and intervene promptly to manage blood sugar and improve the recovery outcomes.

## 5. Conclusions

This research highlights the complex interplay between metabolic and inflammatory pathways in COVID-19 and underscores the potential benefits of early and targeted interventions. In addition to blood glucose at T0, HOMA-IR and hs-CRP significantly contribute to predicting blood glucose at T4 and T12; however, the TyG index does not have additional predictive power compared to these factors.

The study emphasizes the need for a multifaceted approach to managing COVID-19 patients, where understanding and monitoring the glycemic control play a central role. Tailoring interventions based on markers like blood glucose levels, HOMA-IR, and hs-CRP could lead to better outcomes. The findings also suggest that not all commonly used markers (like the TyG index) are universally applicable, highlighting the importance of context-specific research.

Additionally, the inflammatory marker hs-CRP was found to predict worse glycemic control in COVID-19 patients, reflecting how inflammation interacts with metabolic processes. On the other hand, markers like the TyG index, though beneficial in non-COVID-19 populations for predicting insulin resistance, do not hold the same predictive power in the context of COVID-19. This underscores the importance of context-specific research and intervention strategies.

In summary, COVID-19 exacerbates metabolic and inflammatory disturbances, making early and targeted interventions essential to managing glycemic control and mitigating the risk of complications like DM in post-COVID-19 recovery.

These findings underscore the intricate relationship between metabolic factors, inflammation, and glycemic control, particularly post-COVID-19 recovery. Monitoring and managing these parameters can provide valuable insights for predicting and mitigating the risk of DM onset and managing the blood glucose levels in post-COVID-19 patients. By leveraging this knowledge, healthcare providers can develop more effective, personalized treatment plans to improve patient outcomes.

## Figures and Tables

**Table 1:** Differences between Glyc-T0 and Glyc-T4 and Glyc-T0 and Glyc-T12.

	Glyc-T0	Glyc-T4	Glyc-T12
Mean	104.8 ± 33.7	116.2 ± 34.45	119.3 ± 33.1
Pearson correlation with Glyc-T0		0.865	0.770
P(T ≤ t) two-tail		9.25 × 10^−12^	1.12 × 10^−11^

Glyc-T0—initial glycemia; Glyc-T4—glycemia at the 4-month control; Glyc-T12—glycemia at the 12-month control.

**Table 2:** Pairwise correlation between the studied parameters.

	Glyc-T12	Glyc-T4	Glyc-T0	TyG	HOMA-IR	hs-CRP T0	W/H	BMI	TC	TG	HDLc	LDLc
Glyc-T12	1											
Glyc-T4	**0.949**	1										
Glyc-T0	**0.770**	**0.865**	1									
TyG	**0.418**	**0.430**	**0.444**	1								
HOMA-IR	**0.617**	**0.581**	**0.340**	0.170	1							
hs-CRP T0	**0.539**	**0.493**	0.227	0.220	**0.638**	1						
W/H	0.100	0.057	0.001	−0.063	0.030	0.041	1					
BMI	0.150	0.108	0.066	−0.016	0.089	−0.003	0.294	1				
TC	−0.145	−0.143	−0.111	0.217	−0.092	−0.077	0.007	−0.067	1			
TG	0.015	−0.012	−0.044	**0.828**	−0.040	0.073	−0.114	−0.076	**0.376**	1		
HDLc	−0.120	−0.117	0.015	−0.407	−0.119	−0.195	−0.094	0.019	0.186	−0.362	1	
LDLc	−0.135	−0.121	−0.090	0.028	−0.075	−0.084	0.130	−0.016	**0.790**	0.108	0.016	1

Glyc-T12—glycemia at the 12-month control; Glyc-T4—glycemia at the 4-month control; Glyc-T0—initial glycemia; TyG—Triglyceride/Glucose index; HOMA-IR—Homeostasis Model Assessment for Insulin Resistance; hs-CRP T0—high-sensitivity C-reactive protein at T0; W/H—waist/hip ratio; BMI—body mass index; TC—total cholesterol; TG—triglyceride; HDLc—HDL-cholesterol; LDLc—LDL-cholesterol. Bold values highlight important findings.

**Table 3:** Multiple regression—prediction of glycemia dependence on all factors at T4 and T12.

	Glyc-T4		Glyc-T12	
R	0.933		0.886	
	Coefficients	*p*-Value	Coefficients	*p*-Value
Intercept	29.79	0.673	−51.60	0.557
Glyc-T0	0.792	**3.72 × 10^−21^**	0.580	**5.04 × 10^−10^**
TyG	−5.828	0.720	10.01	0.621
HOMA-IR	1.659	**2.05 × 10^−5^**	2.026	**2.77 × 10^−5^**
hs-CRP T0	0.293	**4.14 × 10^−5^**	0.338	**0.00013**
W/H	18.11	0.368	38.84	0.121
BMI	0.157	0.319	0.305	0.121
TC	0.022	0.651	0.0053	0.932
TG	0.0075	0.835	−0.0076	0.865
HDLc	−0.214	0.069	−0.071	0.623
LDLc	−0.038	0.426	−0.042	0.474

Glyc-T4—glycemia at the 4-month control; Glyc-T12—glycemia at the 12-month control; Glyc-T0—initial glycemia; TyG—Triglyceride/Glucose index; HOMA-IR—Homeostasis Model Assessment for Insulin Resistance; hs-CRP T0—high-sensitivity C-reactive protein at T0; W/H—waist/hip ratio; BMI—body mass index; TC—total cholesterol; TG—triglyceride; HDLc—HDL-cholesterol; LDLc—LDL-cholesterol. Bold values highlight important findings.

**Table 4:** Multiple linear regression allowing for the estimation of Glyc-T4 and Glyc-T12 dependence on metabolic factors—selected by prediction significance.

	Glyc-T4		Glyc-T12	
R	0.929		0.876	
	Coefficients	*p*-Value	Coefficients	*p*-Value
Intercept	17.99	6.17 × 10^−6^	33.56	1.19 × 10^−10^
Glyc-T0	0.767	**9.53 × 10^−46^**	0.618	**4.82 × 10^−28^**
HOMA	1.713	**8.82 × 10^−6^**	2.082	**1.61 × 10^−5^**
hs-CRP T0	0.313	**8.63 × 10^−6^**	0.362	**3.76 × 10^−5^**

Glyc-T4—glycemia at the 4-month control; Glyc-T12—glycemia at the 12-month control; Glyc-T0—initial glycemia; HOMA—Homeostasis Model Assessment; hs-CRP T0—high-sensitivity C-reactive protein at T0. Bold values highlight important findings.

**Table 5:** Multiple linear correlation allowing for the estimation of Glyc-T4 and Glyc-T12 dependence on conventional diabetes risk factors.

	Glyc-T4		Glyc-T12	
R	0.204		0.244	
	Coefficients	*p*-Value	Coefficients	*p*-Value
Intercept	114.6	0.048	85.72	0.120
W/H	16.27	0.762	39.45	0.443
BMI	0.444	0.293	0.572	0.155
TC	−0.027	0.837	−0.024	0.846
TG	−0.006	0.883	0.0074	0.848
HDLc	−0.295	0.316	−0.245	0.382
LDLc	−0.066	0.603	−0.082	0.496

Glyc-T4—glycemia at the 4-month control; Glyc-T12—glycemia at the 12-month control; W/H—waist/hip ratio; BMI—body mass index; TC—total cholesterol; TG—triglyceride; HDLc—HDL-cholesterol; LDLc—LDL-cholesterol.

## Data Availability

The data presented in this study are available on reasonable request from the corresponding author.

## Abbreviations

AIRglucose	Acute insulin response to glucose
ALP	Alkaline phosphatase
ALT	Alanine aminotransferase
ARDS	Acute respiratory distress syndrome
AST	Aspartate aminotransferase
BMI	Body mass index
BMP	Basic metabolic panel
BUN	Blood urea nitrogen
COVID-19	Coronavirus disease
DM	Diabetes mellitus
eGFR	Estimated glomerular filtration rate
Glyc-T0	Initial glycemia
Glyc-T4	Glycemia at the 4-month control
Glyc-T12	Glycemia at the 12-month control
HbA1c	Glycated hemoglobin
HDLc	HDL-cholesterol
HOMA-IR	Homeostasis Model Assessment of Insulin Resistance
hs-CRP	High-sensitivity C-reactive protein
LDH	Lactate dehydrogenase
LDLc	LDL-cholesterol
MLR	Monocyte/lymphocyte ratio
MPV	Mean platelet volume
PCR	Polymerase chain reaction
SARS-Cov-2	Severe acute respiratory syndrome coronavirus 2
TC	Total cholesterol
TG	Triglyceride
TyG	Triglyceride/Glucose
W/H	Waist/hip ratio
